# Effects of Decamethylcyclopentasiloxane on Reproductive Systems in Female Rats

**DOI:** 10.3390/toxics11040302

**Published:** 2023-03-25

**Authors:** Jimin Lee, Kangmin Kim, Seon-Mi Park, Jin-Sook Kwon, Eui-Bae Jeung

**Affiliations:** 1 College of Veterinary Medicine, Chungbuk National University, Chengju 28644, Republic of Korea; 2 AevisBio, Inc., Deajeon 34141, Republic of Korea

**Keywords:** decamethylcyclopentasiloxane, developmental toxicity, reproductive toxicity

## Abstract

The female reproductive system becomes fertile through the action of hormones involved in the hypothalamic-pituitary-ovarian axis. On the other hand, estrogen-like endocrine disruptors released into the environment come into contact with humans by various routes and affect the reproductive system. Exposure to these chemicals can cause problems with the reproductive process, from egg ovulation to implantation, or cause female reproductive diseases. These reproductive problems cause infertility. Decamethylcyclopentasiloxane (D5) is used for lubrication in silicone polymers, households, and personal care products. In the case of D5, it is discharged through factory wastewater and can bioaccumulate. Therefore, it accumulates in the human body. In this study, D5 was administered orally for four weeks to determine the effects of D5 on the reproductive process. As a result, D5 increases the number of follicles in the ovary and suppresses the expression of genes related to the growth of follicles. In addition, it increases the gonadotropin hormone, inducing estradiol enhancement and progesterone reduction. Because of these changes in the reproductive system when exposed to D5, the industry should reconsider using D5.

## 1. Introduction

The female reproductive system becomes fertile through the action of various hormones involved in the hypothalamic-pituitary-ovarian (HPO) axis [1]. The action of the gonadotropin-releasing hormone secreted by the hypothalamus stimulates the pituitary gland to promote the secretion of the luteinizing hormone (LH) and the follicle-stimulating hormone (FSH), which are types of the gonadotropin hormone (GTH) [2]. GTH acts on the ovary to increase the secretion of steroid hormones, including estradiol (E2) and progesterone (P4) [3]. Enhanced steroid hormones inhibit the activity of the hypothalamus and pituitary gland [4].

The role of hormones in reproduction is very important, and changes in the HPO axis are caused by hormones produced in vivo. The types of exogenous hormones in the environment are diverse and have several effects. In the case of chemicals used mainly in factory processes, even if the product is not directly used, it is discarded into the environment through wastewater and exposed to humans [5,6,7]. Therefore, various chemicals present in the environment have biomagnification and accumulate in top predators [8,9,10].

Among the endocrine-disrupting chemicals (EDC), estrogenic chemicals cause direct damage to female reproductive organs [11,12]. The effects can cause disease of the reproductive organs, leading to infertility, such as polycystic ovary syndrome (PCOS) and endometriosis (EMS). PCOS is a common disease affecting 5 to 18% of women worldwide. This causes symptoms, such as an excess of androgens, anovulation, and polycystic ovaries, which lead to various complications [13,14]. EMS is also a disease in many women, in which the endometrium attaches to the extrauterine tissue and proliferates. This symptom deforms the pelvic structure and causes pain due to adhesion to a wide range of tissues [15,16].

The causes of infertility due to exposure to these chemicals include the anti-müllerian hormone (*Amh*) in the ovary, Spermatogenesis and oogenesis specific basic helix-loop-helix 2 (*Sohlh2*), Kit ligand (*Kitlg*), and Head box l2 (*Foxl2*). The changes in the expression of genes affect follicle development and cause problems with ovulation [17] or failure of endometrial proliferation due to changes in the genes of the heart. Neural crest derivatives expressed 2 (*Hand2*), Fk506 binding protein 4 (*Fkbp4*), and Homeobox a 10 (*Hoxa10*) in the uterus can cause implantation difficulties [18]. Problems with the synthesis and secretion of GTH and steroid hormones can cause infertility [19,20]. Increases in GTH are associated with steroidogenic acute regulatory protein (*Star*); Cytochrome P450 family 11 subfamily A member 1 (*Cyp11a1*); Hydroxy-delta-5-steroid dehydrogenase, 3 beta-and steroid delta isomerase 1 (*Hsd3b1*) and Cytochrome P450 family 19 subfamily A member 1 (*Cyp19a1*) genes increases steroid hormone production [21]. The expression of receptors that respond to the secretion of hormones increases, and the same strong activity occurs when the hormones increase [22]. These various causes can lead to problems with the reproductive system in females related to pregnancy.

Decamethylcyclopentasiloxane (D5; CAS number. 541-02-6), a type of cyclic volatile methyl siloxane (cVMS) used in this study, has been produced commercially since the 1940s. This compound has also been used in silicone polymers, household products, and personal care products [23]. D5, used in producing various products for lubrication purposes [24], has been released into the environment through factory processes [25,26,27]. D5 bioaccumulates, and more research is needed to examine the effect.

Female pregnancy failure is also caused by a problem in the development of the embryo. After the implantation of the fertilized egg, the cardiovascular system is first formed during the development of the mammalian embryo [28]. Problems at this stage make it difficult to deliver blood to the tissue, leading to embryonic death in utero [29]. This experiment aimed to determine whether D5 is toxic at the developmental stage at the in vitro level using the embryoid body test (EBT), which confirms the cardiac differentiation capacity of embryos [30,31,32]. In this study, the effects of D5 on the reproduction process in females were examined.

## 2. Materials and Methods

### 2.1. Chemicals

Decamethylcyclopentasiloxane (D5; 444278, Sigma-Aldrich, St. Louis, MO, USA) was dissolved in 100% ethanol (100983, Merck, Rahway, NJ, USA) to prepare a stock solution, and dissolved in corn oil (C8267, Sigma-Aldrich, St. Louis, MO, USA).

### 2.2. Animal and Chemical Treatments

All experiments were approved by the Chungbuk National University Institution of Animal Care and Use Committees under Ethics Approval No. CBNUA-1556-21-01 for animal care and use for scientific purposes. The 47-week-old Sprague Dawley (SD) rats used in the experiment were purchased from Samtaco (Osan, Republic of Korea). The rats were housed in an air-filtered Semi-SPF that maintained a temperature and humidity of 22 ± 2 °C and 50 ± 10%, respectively, to maintain animal health. They were fed an AIN-76A rodent diet (D10001, Research Diets, New Brunswick, NJ, USA) with sterilized water.

According to the Scientific Committee on Consumer Safety (SCCS) in 2016, the LOAEL and NOAEL for the oral route of D5 were reported to be 100 mg/kg bw/day and 25 mg/kg bw/day [33]. Based on this, the chemical was administered orally. Corn oil was used as a vehicle, and D5 was treated daily at 1 mg/kg, 10 mg/kg, 100 mg/kg, or 200 mg/kg at 5 kg/mL.

### 2.3. Cell Culture

Mouse fibroblast cells (3T3-L1, CL-173) and embryonic stem cells (mES; ES-E14TG2a, CRL-1821) were purchased from the American Type Culture Collection (ATCC; Manassas, VA, USA). The 3T3-L1 was cultured in media containing a Dulbecco-modified eagle medium (DMEM; LM001, Welgene, Gyeongsan, Republic of Korea), 10% fetal bovine serum (FBS; S1480, Gibco, Grand Island, NY, USA), 100 U/mL penicillin, and 100 mg/mL streptomycin (L0022, Biowest, Nuaillé, French).

The isolated mouse embryonic fibroblast cells (mEF) were treated with mitomycin c (10107409001, Roche, Basel, Switzerland) and used as feeder cells. The media for maintaining mES cells in an undifferentiated state included DMEM/F-12 (11320033, Gibco, Grand Island, NY, USA), 10% FBS (16000044, Gibco, Grand Island, NY, USA), 1% MEM non-essential amino acids solution (11140050, Gibco, Grand Island, NY, USA), 100 U/mL penicillin and 100 mg/mL streptomycin, 5 μg/mL plasmocin prophylactic (ant-mpp, Invitrogen, Waltham, MA, USA), 10^−4^ M 2-mercaptoethanol (21985023, Gibco, Grand Island, NY, USA), and 10 ng/mL mouse leukemia inhibitory factor (mLIF; ESG1107, Sigma-Aldrich, St. Louis, MO, USA). mES was differentiated into the myocardium when cultured in undifferentiated media with 15% FBS, except for mLIF.

The cells were cultured in an incubator (MCO-18AIC, Sanyo, Osaka, Japan) at 37 °C and 5% CO_2_. The wash process was performed twice with Dulbecco’s phosphate-buffered saline (D-PBS; LB001-02, Welgene, Gyeongsan, Republic of Korea), and 0.05% trypsin-EDTA (LS015-09, Welgene, Gyeongsan, Republic of Korea) was used to separate the cells from the dish.

### 2.4. Embryoid Body Test (EBT)

The cell viability was measured by seeding 800 cells in 50 μL/well of maintenance media into a 96-well plate. D5 was treated at a concentration of 10^−3^–10^−11^ M so that the volume per well was 200 μL. After culturing for three days, the plate was washed twice with D-PBS and 10 μL of EZ-cytox (EZ-500, Dogenbio, Seoul, Republic of Korea), and each well was treated with 90 μL of media. The plate was incubated for 1 h, and the absorbance at a wavelength of 450 nm was measured.

EB formation was performed using the hanging drop method. In a 90 mm petri dish, up to 85 drops of differentiated media were added, including 800 cells in each 20 μL drop. The samples were cultured for 3 days and collected in a 35 mm petri dish. Images were taken at 100× magnification with a phase contrast microscope (IX71, Olympus, Tokyo, Japan). For the eight images obtained, the size of the EB area was measured using Image J (NIH, Bethesda, MD, USA).

### 2.5. Tissue Fixation and Section

Immediately after obtaining the ovary tissue, it was fixed in 10% neutral buffered formalin and embedded in paraffin. A total of 5 serial sections were performed in units of 60 μm. The sections (6 μm thick) were stained with hematoxylin and eosin.

### 2.6. RNA Extraction, Complementary DNA Synthesis, and Quantitative Real-Time PCR

For total RNA extraction, the obtained tissue was placed in containing beads Trizol (15596026, Invitrogen, Waltham, MA, USA) and homogenized using bead ruptor 12 (OMNI, Kennesaw, GA, USA). Chloroform was added at one-fifth of the trizol level and centrifuged at 14,000 rpm for 10 min. The supernatant was obtained by adding 100% ethanol at 1.5 times the volume of the supernatant and transferred to a spin column. The mixture was centrifuged at 8000 rpm for 15 s. RW1 buffer (700 μL, 1053394, Qiagen, Hilden, Germany) was added and centrifuged. RPE buffer (350 µL, 1018013, Qiagen, Hilden, Germany) was added, and the centrifugation was run twice. The membrane was dried by running the spin column at 14,000 rpm for 1 min. For elution, 100 μL of DNase RNase-free water was added to the membrane. After 30 **s**, elution proceeded at 14,000 rpm for 1 min.

cDNA was synthesized using an iscript cDNA synthesis kit (BRI70-8891, Bio-Rad, Hércules, CA, USA) from 1 μg RNA. The synthesized cDNA was diluted 1:50 in distilled water.

A Quantstudio 3 real-time PCR instrument (applied biosystems, Foster City, CA, USA) was used to measure the quantitative real-time PCR (qPCR). In a qPCR cocktail, 6.25 μL of 2× prime q-master mix (with SYBR green I), 0.25 μL of 50× ROX dye (Q-9212, GENETBIO, Daejeon, Republic of Korea), 1 μL of forward and reverse primer (200 μM each), 3 μL of distilled water, and 2 μL of cDNA were included. Table 1 lists the primer information. The qPCR cycle consisted of 40 cycles, followed by 95 °C for 30 s for denaturation, 58 °C for 30 s for annealing, and 72 °C for 30 s for elongation. The fluorescence was measured at the end of each PCR stage. The ΔΔCT values were analyzed using the Quantstudio design and analysis software.

### 2.7. Blood Collection and Serum Separation

For serum biochemical analysis, blood was collected by cardiac puncture after sacrifice. The blood was incubated overnight at 4 °C and centrifuged at 4000× *g* for 10 min. The serum was immediately stored in a −70 °C deep freezer. The serum concentrations of LH (CEA441Ra), FSH (CEA830Ra), and AMH (CEA228Ra) were measured using a Cloud-clone (Katy, TX, USA) ELISA kit.

### 2.8. Statistical Analyses

The data were visualized and analyzed statistically using GraphPad Prism 8 for Windows (GraphPad Software, San Diego, CA, USA). The values in the graph were reported as means ± standard deviations (SD). An ordinary one-way analysis of variance (ANOVA) was used for comparison, and statistical significance was considered *p*-value < 0.05.

## 3. Results

### 3.1. Developmental Toxicity of D5

**Figure 1:**
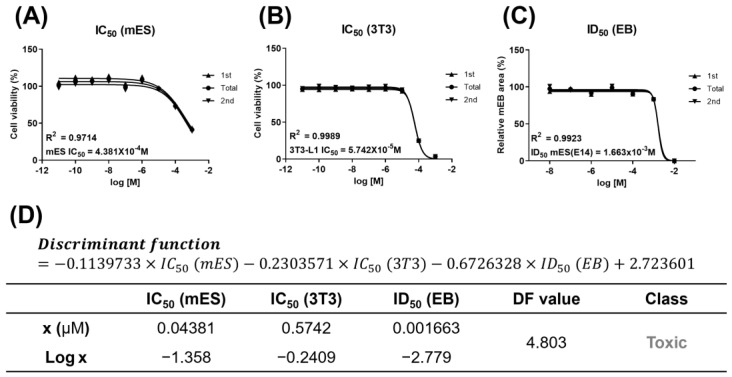
Developmental toxicity of D5 using EBT. Measurement of cell viability using mES and 3T3-L1 and half inhibitory concentration for EB size using mES: (**A**) IC_50_ values of mES; (**B**) IC_50_ values of 3T3-L1; (**C**) ID_50_ values of mES; (**D**) table of EBT-derived values. All values were expressed as mean, and the R^2^ value refers to the difference between the first value and the second value.

When treated with D5, the cell viability of the two cell lines, mES and 3T3-L1, had IC_50_ values of 0.04381 μM (mES) and 0.5742 μM (3T3), respectively (Figure 1A–D). In addition, the half inhibitory concentration of the EB size, ID_50_, was 0.001663 μM (Figure 1C,D). A value of 4.803 was obtained by substituting this derived value into the previously developed discriminant function (Figure 1D). D5 had developmental toxicity because the discriminant function score was higher than the reference value of −0.667.

### 3.2. Organ Weights and Polycystic Ovarian Morphology Induced by Long-Term Exposure to D5

#### 3.2.1. Polycystic Ovarian Morphology Induced by D5

**Figure 2:**
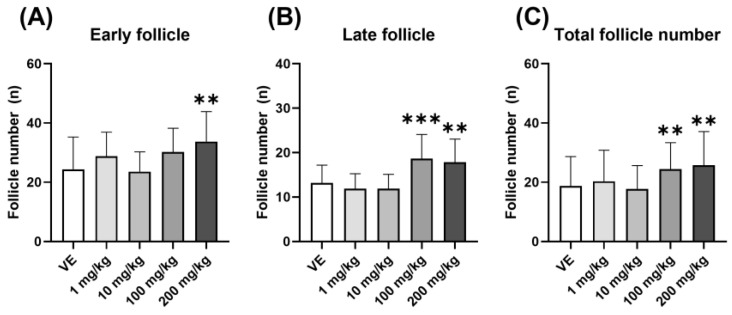
Number of follicles in the ovary of rats exposed to D5. The comparison of the number of ovary follicles in the two stages and the total number of ovary follicles were confirmed: (**A**) number of early follicles including primordial follicle, primary follicle, and secondary follicle; (**B**) number of late follicles including antral follicle and preovulation follicle; (**C**) the total number of follicles including early follicle and late follicle. The values are expressed as means ± SD. ** *p* < 0.01 and *** *p* < 0.001 vs. vehicle. VE—vehicle, *N* = 9~10.

The number of follicles located at two growth stages was measured to confirm follicle development in the ovary. Upon treatment with D5, the number of early follicles, including primordial, primary, and secondary follicles, increased in the 200 mg/kg group (Figure 2A). In addition, the number of late follicles, including antral and preovulation follicles, increased in the 100 mg/kg and 200 mg/kg groups (Figure 2B). Therefore, the total number of follicles increased in the 100 mg/kg and 200 mg/kg groups (Figure 2C). This confirmed that the number of follicles in the ovary increased when exposed to D5. An increase in follicles in the ovary also means a representative phenotype of PCOS, and ovulation is not taking place due to the accumulation of follicles.

#### 3.2.2. Weight of Uterus and Ovary Compared to Body Weight

**Figure 3:**
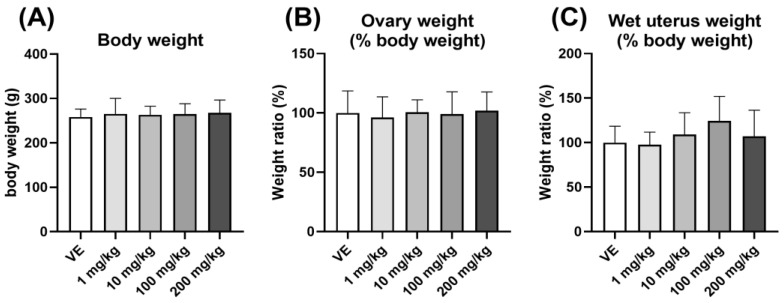
The body weight and organ weight of rats exposed to D5. (**A**) Body weights of rats; (**B**) ratio of ovary weight to body weight; (**C**) ratio of uterus weight to body weight. The values are expressed as means ± SD. VE—vehicle, *N* = 9~10.

The body weight was measured, and the % body weight of the ovary and uterus was checked to confirm the induction of obesity in rats exposed to D5. No significant change in body weight (Figure 3A) and ovary and uterus weight (Figure 3B,C) were observed. There was no change in organ weight when exposed to D5, suggesting that it did not induce obesity.

### 3.3. Hormonal Changes in Serum When Exposed to D5

#### 3.3.1. Changes in Gonadotropin Hormones and Anti-Müllerian Hormone

**Figure 4:**
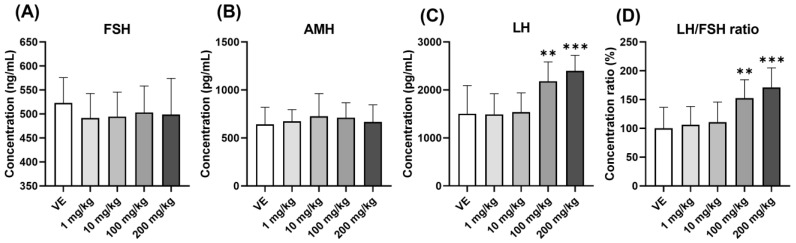
Changes in the concentration of the gonadotropin hormone and the anti-müllerian hormone in the serum. Confirmation of hormones in the serum of rats exposed to D5 for four weeks using ELSIA: (**A**) FSH concentration in serum; (**B**) AMH concentration in serum; (**C**) LH concentration in serum; (**D**) concentration ratio of LH and FSH in serum. The values are expressed as means ± SD. ** *p* < 0.01 and *** *p* < 0.001 vs. vehicle. VE—vehicle, *N* = 9~10.

The serum hormone concentrations were checked to investigate the cause of the increased follicle formation in the ovary. When exposed to D5, there was no significant change in FSH, a hormone that stimulates follicle formation in the serum (Figure 4A). AMH, a marker of PCOS, also showed no significant change (Figure 4B). On the other hand, there was an increase in LH in the 100 mg/kg and 200 mg/kg groups (Figure 4C). The LH/FSH ratio, another known marker of PCOS, increased (Figure 4D). The possibility of affecting the target organ was confirmed by changing the hormone concentration. Through the increase in LH in rats exposed to D5, D5 affected the pituitary gland and increased GTH secretion.

#### 3.3.2. Changes in Steroid Hormones

**Figure 5:**
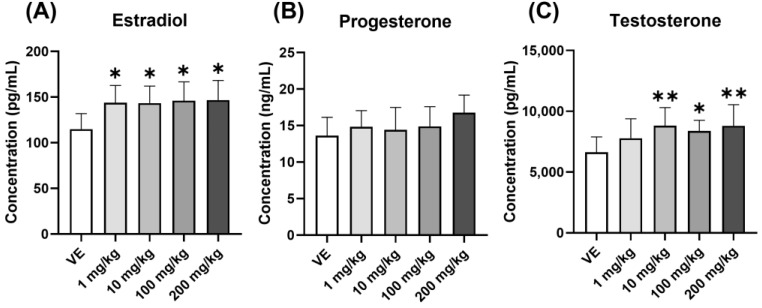
Changes in the concentration of steroid hormone in the serum. Confirmation of hormones in the serum of rats exposed to D5 for four weeks using ELSIA: (**A**) Estradiol concentration in serum; (**B**) progesterone concentration in serum; (**C**) testosterone concentration in serum. The values are expressed as means ± SD. * *p* < 0.05 and ** *p* < 0.01 vs. vehicle. VE—vehicle, *N* = 9~10.

The secretion of steroid hormones was changed by the increased secretion of LH. Exposure to D5 increased serum E2 concentrations (Figure 5A). On the other hand, the concentration of P4 did not change (Figure 5B), and the concentration of testosterone increased (Figure 5C). As a result, all steroid hormones increased due to the increase in GTH, and it was necessary to confirm what problems occurred in the organs of the female reproductive system.

### 3.4. Expression Levels of the Gonadotropin Hormone Receptor and the Steroid Hormone Receptor in Reproductive Organs

**Figure 6:**
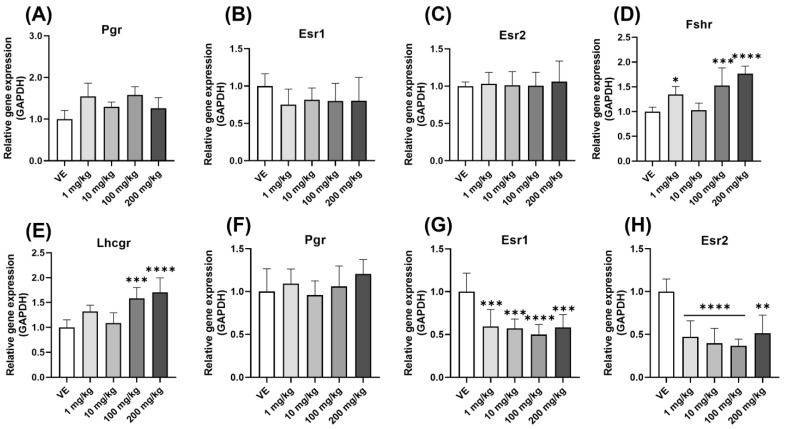
Changes in steroid hormone receptor expression by D5 exposure. Gene expression was confirmed using qPCR in the ovary and uterus of rats exposed to D5 for four weeks: (**A**) *Pgr* expression level in the ovary; (**B**) *Esr1* expression level in the ovary; (**C**) *Esr2* expression level in the ovary; (**D**) *Fshr* expression level in the ovary; (**E**) *Lhcgr* expression level in the ovary; (**F**) *Pgr* expression level in the uterus; (**G**) *Esr1* expression level in the uterus; (**H**) expression level of *Esr2* in the uterus. The values are expressed as means ± SD. * *p* < 0.05, ** *p* < 0.01, *** *p* < 0.001 and **** *p* < 0.0001 vs. vehicle. VE—vehicle; Fshr—follicle-stimulating hormone receptor, Lhcgr—luteinizing hormone/choriogonadotropin receptor; *N* = 9~10.

As in the previous results, receptor expression also increased with GTH. Steroid hormones act mainly by binding to receptors. Therefore, the changes in the expression level of steroid hormone receptors were observed when exposed to D5. First, there was no change in the levels of Pgr, Esr1, and Esr2 expression in the ovary (Figure 6A–C). The expression levels of Fshr (Figure 6D) and Lhcgr were also increased (Figure 6E). Second, there was no significant change in Pgr in the uterus (Figure 6F), and the levels of Esr1 and Esr2 expression decreased (Figure 6G,H). As a result, the effect of Fshr and Lhcgr in the ovary increased, and the effect of E2 targeting the uterus increased.

### 3.5. Comparison of Gene Expression in Ovary

#### 3.5.1. Steroidogenesis Gene

**Figure 7:**
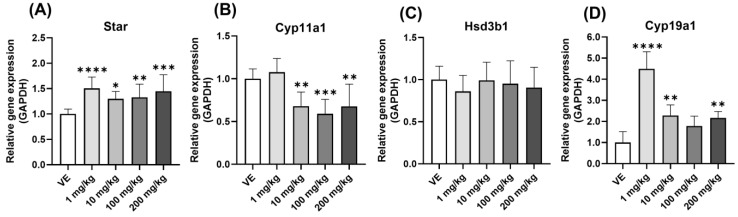
Changes in the expression level of ovary steroid hormone formation upon exposure to D5. Gene expression confirmed by qPCR in the ovary of rats exposed to D5 for four weeks: (**A**) Star expression level; (**B**) Cyp11a1 expression level; (**C**) Hsd3b1 expression level; (**D**) Cyp19a1 expression level. The values are expressed as means ± SD. * *p* < 0.05, ** *p* < 0.01, *** *p* < 0.001 and **** *p* < 0.0001 vs. vehicle. VE—vehicle, *N* = 9~10.

Figure 6 shows the increased effect of steroid hormone, and changes in steroidogenesis in the ovary were checked to find the cause of their increased effect. The changes in the genes that play important roles in synthesizing steroid hormones were identified. The level of Star expression was increased in all groups treated with D5 (Figure 7A). On the other hand, the level of Cyp11a1 expression decreased (Figure 7B), and there was no significant change in the level of Hsd3b1 expression (Figure 7C). Cyp19a1 showed a sharply increasing pattern in the 10 mg/kg group (Figure 7D). Therefore, the amount of steroid hormone synthesis increased, and the conversion to E2 also increased.

#### 3.5.2. Folliculogenesis Gene

**Figure 8:**
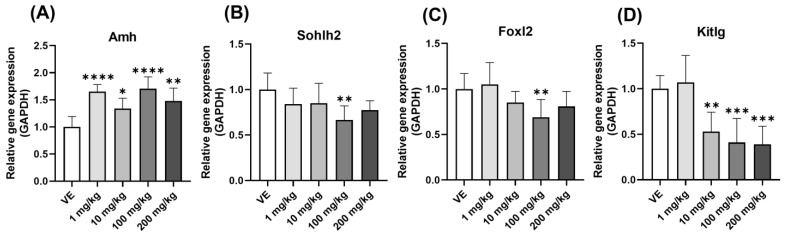
Changes in the expression level of genes involved in ovarian follicle formation when exposed to D5. Gene expression confirmed by qPCR in the ovary of rats exposed to D5 for four weeks: (**A**) Amh expression level; (**B**) Sohlh2 expression level; (**C**) Foxl2 expression level; (**D**) Kitlg expression level. The values are expressed as means ± SD. * *p* < 0.05, ** *p* < 0.01, *** *p* < 0.001 and **** *p* < 0.0001 vs. vehicle. VE—vehicle, *N* = 9~10.

The steroid hormone is produced in the ovary and affects the hypothalamus, pituitary gland, ovary, and uterus. The cause of histological changes in the ovary was examined by measuring the expression levels of genes that play an important role in follicle formation. First, in the case of Amh, the expression level increased effectively (Figure 8A), and Sohlh2, Foxl2, and Kitlg showed a decreasing pattern (Figure 8B–D). As a result, the ovaries exposed to D5 had developmental problems and did not ovulate normally. Anovulation in this ovary causes menstrual irregularities and infertility.

### 3.6. Confirmation of Implantation-Related Gene Expression in the Uterus

**Figure 9:**
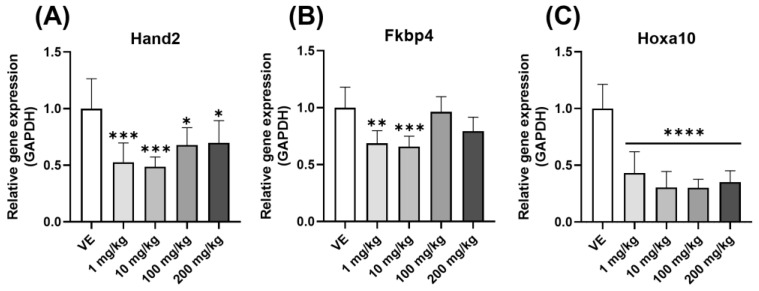
Changes in the expression levels of genes associated with implantation in the uterus when exposed to D5. Gene expression confirmed by qPCR in the uterus of rats exposed to D5 for four weeks: (**A**) Hand2 expression level; (**B**) Fkbp4 expression level; (**C**) Hoxa10 expression level. The values are expressed as means ± SD. * *p* < 0.05, ** *p* < 0.01, *** *p* < 0.001 and **** *p* < 0.0001 vs. vehicle. VE—vehicle, *N* = 9~10.

The steroid hormones secreted from the ovary influence the target organ, which is the uterus. The expression levels of the genes related to the sensitivity of uterine epithelium were confirmed. The levels of Hand2, Fkbp4, and Hoxa10 expression decreased (Figure 9A–C). Hence, D5 can cause problems in the implantation process. Similar to anovulation, this can cause infertility.

## 4. Discussion

D5 is used in cosmetics and household products and is discharged to wastewater during manufacturing. The main cosmetics users are women, who are more exposed to D5 than men. Therefore, the effects of D5 on women were confirmed. A previous study reported that D4, which has a similar structure to D5, affects the female reproductive system [34,35,36]. Because of the similar structure, D5 was also studied with the expectation that it would influence the female reproductive system. Reproduction proceeds in the order of egg ovulation, fertilization, and implantation [37]. Among them, the effects of D5 on ovulation and implantation were studied.

When radioactively labeled D5 is administered orally, it is excreted in the urine of rats as various metabolites [38]. In another study, when two concentrations of D5 were administered orally, urinary recovery was 4.39% and 8.15% for the high and low doses, respectively [39]. This means that D5 is saturated in the metabolic process in the body. Therefore, this study focused on the effects of D5 rather than the effect of the concentration of D5.

The egg is ovulated through follicle development. At this time, various transcription factors act on the growth of the oocyte. *Kitlg* is in early oocytes and granulosa cells and affects follicle development [40]. *Kitlg* and c-KIT signaling are essential for oocyte survival, and they activate the PI3K pathway when combined [41]. Activation of PI3K means an increase in cell proliferation [42]. Moreover, *Foxl2* is involved in most ovarian development and function in granulosa cells [43]. Defective *Foxl2* stops the squamous to the cuboidal transition of granulosa cells, preventing secondary follicle development [44]. In addition, *Sohlh2* was expressed predominantly in early primordial and primary follicles. A deficiency of this gene caused oocyte and follicle loss [45]. In this study, *Kitlg* and *Foxl2* were decreased by the D5 treatment, which inhibited granulosa cell differentiation, indicating that problems occurred in early follicle development. In addition, the decrease in *Sohlh2* indicates that D5 can cause the premature depletion of oocytes.

A typical egg is fertilized and implanted into the endometrium. Among the several genes contributing to endometrial receptivity, *Hand2* suppresses several fibroblast growth factors to increase receptivity in the uterus [46]. The inhibition of *Hand2* causes a decrease in endometrial receptivity caused by abnormal endometrial proliferation [47]. *Hoxa10* also plays an important role in the implantation, decidualization, and immune regulation of the adult uterus. Low expression of *Hoxa10* impairs endometrial differentiation [48]. In addition, *Fkbp4* must bind to *Pgr* for transcript activation during implantation and is a downstream gene of *Hoxa10* [49,50]. Endometrium proliferation decreased when the expression of *Hand2*, *Hoxa10*, and *Fkbp4* was reduced in rats expressed to D5. These problems lead to decreased endometrial receptivity, resulting in infertility.

The problems with the reproduction process may have been caused by dysfunction of the HPO axis. An increase in LH causes problems with follicle maturation and induces abnormal reproductive processes [51]. The resulting increase in the LH/FSH ratio also means an increase in androgen production in theca cells [52]. Exposure to D5 caused problems with follicle maturation and affected ovarian development by stimulating corpus luteum formation.

The secreted hormones combine with hormone receptors and are activated [53]. Therefore, the change in the receptor expression level indicates the action of the hormone. The *Lhcgr* gene, an LH receptor, is expressed in granulosa cells [54]. In addition, the FSH receptor, *Fshr*, is a G-protein coupled receptor located in the granulosa cells and controls the processing and movement of steroid hormones [55]. Therefore, the increase in *Lhcgr* and *Fshr* confirmed the increase in steroid hormone production in ovary granulosa cells caused by GTH.

The steroid hormone is synthesized in the ovary, and failure or overexpression of this process significantly affects the reproductive system. Steroidogenesis is a step-by-step process. Star promotes the transport of cholesterol within the mitochondria [56]. The transported cholesterol is converted to pregnenolone by Cyp11a1 [57]. The converted pregnenolone is metabolized by Hsd3b1 to synthesize P4 [58]. Cyp19a1 also converts testosterone to E2 [59]. Through this, Star and Cyp19a1 induction increased E2 expression and decreased Cyp11a1 reduced P4 synthesis. Hence, D5 causes problems in steroidogenesis in the ovary.

Esr expression increased and Pgr expression decreased when pituitary gland cells were exposed to E2 [60]. Changes in the expression level of these steroid hormone receptors could indirectly confirm the effect of E2. Through the above results and changes in steroid hormone receptors, when exposed to D5, the effect of E2 in the body increased due to the decrease in Esr expression in the uterus. Increased expression of steroid hormones causes a variety of problems. Increased E2 causes endometriosis, an estrogen-dependent disease, or increases the risk of breast, uterine, and ovarian cancer. In addition, the increased secretion of testosterone in women increases the incidence of polycystic ovary syndrome.

In addition, developmental toxicity was checked to determine if there were any problems with embryo growth after egg implantation.

The experiment found that exposure to D5 had several negative effects, including increasing GTH levels, decreasing follicle development, and increasing both the number of follicles and steroid hormone expression in the ovary. This study confirmed that exposure to D5 reduces endometrial receptivity and has developmental toxicity. These results suggest that D5 has similar effects to E2, a hormone essential for female reproductive health. These findings imply that D5 exposure disrupts the normal balance of steroid hormones in the body, potentially leading to various health problems. Thus, further discussion is necessary to determine the safety of D5 in products for women and pregnant women by industry.

## 5. Conclusions

Exposure to D5 in the female body increases the number of follicles in the ovary, increases the secretion of LH in the serum and the steroid hormones estradiol and testosterone, and indicates fetal developmental disorders. The limitations of the use of D5 should be considered because there are still many industries that use it.

## Figures and Tables

**Table 1:** qPCR primer information.

Gene Name	Forward (5′ → 3′)	Reverse (5′ → 3′)
*Pgr*	TGGTTCCGCCACTCATCA	TGGTCAGCAAAGAGCTGGAAG
*Esr1*	GACTTGAATCTCCACGATCA	CTTCAAGGTGCTGGATAGAA
*Esr2*	TCCGGCTCTTAGAAAGCTGC	CCCCTCATCCCTGTCCAGAA
*Fshr*	CTTGAAGCGGCAAATCTCTG	GAGCAGGTCACATCAACAAC
*Lhcgr*	CTCACTGAAAACACTGCCCT	ATGGCGGAATAAAGCGTCTC
*Star*	GCGGAACATGAAAGGACTGA	TCCTTGCTGGATGTAGGACA
*Cyp11a1*	GCTTTGCCTTTGAGTCCATC	CATGGTCCTTCCAGGTCTTA
*Hsd3b1*	TGCCACTTGGTCACACTGTCA	CCCTGTGCTGCTCCACTAGTGT
*Cyp19a1*	GGCAAGCACTCCTTATCAAACC	TCCACGTCTCTCAGCGAAAA
*Amh*	CTGGCTGAAGTGATATGGGA	CACAGTCAGCACCAAATAGC
*Sohlh2*	AGCCAGCTCCAGTTGTCTGT	GATGCTGGATGAGGCAGT
*Kitlg*	TTCAAGGACTTCATGGTGGC	GCGGCTTTCCTATTACTGCT
*Foxl2*	TCGCTAAGTTCCCGTTCTAC	GTAATTGCCCTTCTCGAACA
*Hand2*	CCACCAGCTACATCGCCTAC	CTGTCCGGCCTTTGGTTTTC
*Fkbp4*	TTCCAGATCCCACCACATGC	TCCTTGAAGTACACGGTGCC
*Hoxa10*	GCTGGGGTGCACATCATAAA	TGCCTCAAAGTGGCAGTCG
*Gapdh* *	GGCAAGTTCAATGGCACAGT	TGGTGAAGACGCCAGTAGACTC

* Internal control gene.

## Data Availability

Not applicable.
